# Thermal side effects during pulsed field ablation: an analysis using computer modelling

**DOI:** 10.1093/europace/euaf035

**Published:** 2025-02-17

**Authors:** Marcela Mercado-Montoya, Tatiana Gomez-Bustamante, Steven R Mickelsen, Erik Kulstad, Ana González-Suárez, Lawrence J Overzet

**Affiliations:** Bioengineering Department, IN SILICO STEM S.A.S, Calle 52 81-48, Medellín 050035, Colombia; Bioengineering Department, IN SILICO STEM S.A.S, Calle 52 81-48, Medellín 050035, Colombia; Department of Electrophysiology, Scripps Health, La Jolla, CA, USA; Department of Emergency Medicine, University of Texas Southwestern Medical Center, Dallas, TX, USA; BioMIT, Department of Electronic Engineering, Universitat Politècnica de València, València, Spain; Department of Electrical and Computer Engineering, University of Texas at Dallas, Dallas, USA

**Keywords:** Pulsed field energy, Computational analysis, *In silico* modelling, Simulation, Thermal ablation, Atrial fibrillation

## Abstract

**Aims:**

Pulsed field ablation (PFA) is described as non-thermal, but data from oncology and cardiology show thermal effects occur. The specific waveform parameters influencing thermal energy development during PFA are unclear. The aim of this study is to numerically evaluate the thermal effects of PFA on myocardial and oesophageal tissue at various peak voltage conditions.

**Methods and results:**

A three-dimensional computer model of the left atrium quantified thermal effects from PFA at peak voltages of 1, 1.5, and 2 kV. Energy was applied using a bipolar configuration with far-field and symmetry boundaries set as electrically insulating. A monophasic waveform with a 100 μs pulse width and a 1 s gap between pulses was applied for a total of 50 pulses, mimicking clinical conditions. Minimal temperature rise in the oesophagus was observed with 1 kV pulses (214.5 J). At 1.5 and 2 kV (570.3 and 1.23 kJ), temperatures reached 46.3°C and >62°C, respectively, after a single pulse train. These findings suggest that repeated applications could lead to even higher temperatures, especially if good tissue contact is obtained. These results align with data from other medical fields using pulsed field treatments.

**Conclusion:**

Thermal effects from PFA depend on the total energy deposited, with peak voltage being a significant factor. Current commercially available PFA systems have the potential to induce collateral thermal injury with repeated applications of pulsed field energy. This highlights the need for careful monitoring and adjustment of PFA parameters in clinical settings.

## Graphical Abstract

**Graphical Abstract:**
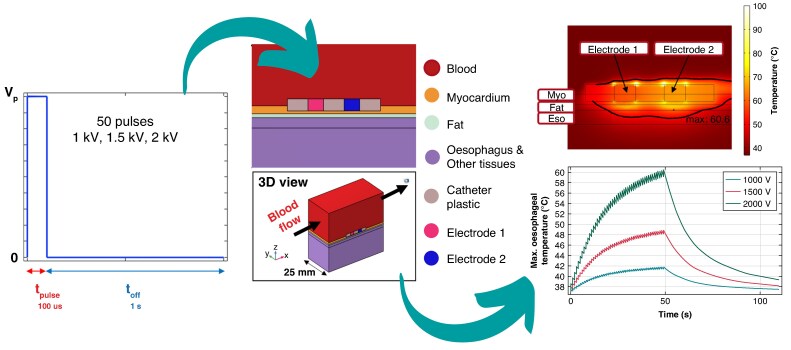

**What's new?**

- Although the mechanism of cell death from pulsed field ablation (PFA) is non-thermal, it is increasingly appreciated that the energy produced from pulsed fields results in thermal energy.
- Recent clinical electrophysiology data have shown luminal oesophageal temperatures as high as 40.3°C from PFA applications, and data from the oncology literature confirm both similar thermal effects, as well as the occurrence of various types of fistulas, from PFA use.
- To further elucidate the physics behind this phenomenon, this study utilized a robust computational model that leverages the efficiencies of mathematical models to determine which factors most affect the thermal impact and the magnitude of this effect.
- The results show that current commercially available systems may induce collateral thermal injury with repeated applications of pulsed field energy.

## Introduction

Pulsed field ablation (PFA) is the result of an impressive evolution in the technology of ablation, beginning with the first use as direct current ablation in the 1980s.^1,2^ Pulsed field ablation has been described as non-thermal, but abundant data exist in oncology applications,^3–5^ and growing data are emerging in cardiology,^6,7^ highlighting that thermal effects are in fact present with PFA. These thermal effects have been shown to result in luminal oesophageal temperatures above the thresholds typically considered to increase the risk for atrio-oesophageal fistula formation.^6^ Although the predominant mechanism of cell destruction with PFA may rely on non-thermal electroporation, the fact that thermal effects are present has prompted experts to recommend that electroporation should in fact not be called ‘non-thermal’.^3^ Preclinical studies have shown that supratherapeutic PFA can induce acute oesophageal lesions, and preclinical healing patterns may not be replicated in patients, so that it may take upwards of several thousands of patients (including those who undergo ablation of persistent atrial fibrillation, where multiple repeat applications may be performed) to truly conclude that fistula formation is not possible.^8^ Finally, in the field of oncology, where PFA (also referred to as irreversible electroporation) has been used to treat solid tumours for well over a decade, thermal effects are well documented, with fistulas occurring in up to 10.6–20% of patients.^4,5,9–12^ As such, we sought to evaluate the thermal effects arising from PFA of myocardial and oesophageal tissue using *in silico* models examining a range of typical peak voltage operating conditions.

## Methods

A three-dimensional computer model of the left atrium and oesophagus was created to quantify the thermal effects from PFA applications. Specific details of the model are described in the work by Mercado-Montoya *et al*.^13^ and are similar to other published work.^14^ Broadly, a three-dimensional model representing the PFA procedure served as the computational framework, which encompassed a catheter featuring two cylindrical electrodes (representing momentary anode and cathode) separated by plastic. The model accounts for the myocardium, pericardium (fat layer), and oesophagus, each with specific thicknesses (1.5, 0.75, and 2 mm, respectively). The catheter is assumed to have full contact with the myocardium.

## Results

**Figure 1:**
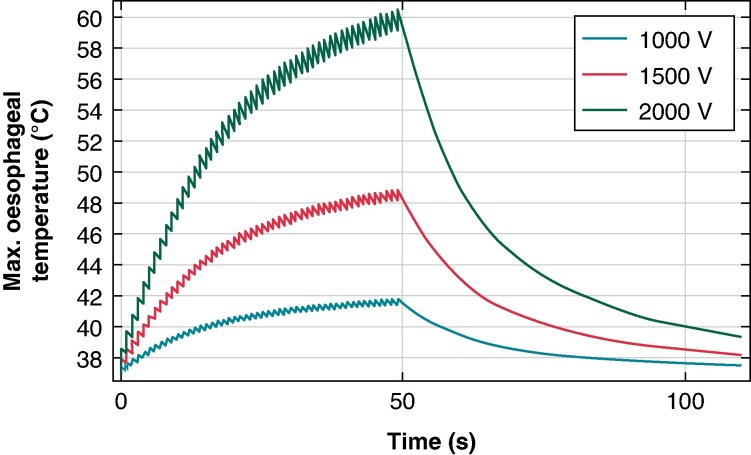
The evolution with time of the maximum temperature in the oesophagus as a function of applied pulse peak voltage.

Minimal temperature rise in the oesophagus was seen with 1 kV pulses (corresponding to 13.4 J input). With 1.5 and 2 kV peak voltages (corresponding to 32.3 and 66.2 J), temperature elevations reaching 46.3°C and >62°C were seen, respectively. These elevations occurred after only a single pulse train of 50 pulses, implying that further elevations in temperature would be seen with subsequent applications. Total energy for 1, 1.5, and 2 kV pulse peak voltages is 214, 570, and 1282 J, respectively. Average energy per pulse for 1, 1.5, and 2 kV pulse peak voltages is 4.28, 11.4, and 26.7 J, respectively. *Figure 1* presents the evolution with time of the maximum temperature in the oesophagus, showing both the rising and falling temperature curves between pulses, as well as the accumulated thermal effect. This effect is most pronounced at 1500 and 2000 V, with an apparent non-linearly increasing behaviour on the thermal damage by increasing the pulse peak voltages. This behaviour has been also seen by other authors in Aycock *et al*.,^15^ Faroja *et al*.,^4^ and van Gemert *et al*.,^3^ supporting the reliability of the numerical results.

## Discussion

These results align well with recent data published in the electrophysiology literature, as well as abundant data in the field of oncology. Faroja *et al*.^4^ measured tissue temperatures in a porcine model using a commercially available system (Nanoknife Tissue Ablation System, AngioDynamics). Using voltage settings of up to 3 kV and pulse durations of 100 µs, they found post-ablation temperatures ranging between 34°C for low-voltage 1500 V applications and 90 pulses to 84°C for high-voltage 2900 V applications and 360 pulses. All combinations of voltage > 2500 V for 90 or more pulses produced temperatures > 50°C, which were associated with gross and histopathologic findings of thermal coagulation.^4^ Dunki-Jacobs et al., and Agnass *et al*. subsequently reported similar findings of considerable heating sufficient to cause thermal damage, while also noting an exacerbating influence from the presence of metal within the ablation field,^5,9^ Mathematical models of oncologic applications have also reported significant thermal effects.^3^

The data presented here stem from a high-quality modelling approach and further reinforce the point that thermal effects occur with PFA, and in particular, in cardiac applications, thermal effects can result in temperature rises sufficiently to raise concern.^6,14^ Clinical validation through extensive patient studies is essential to confirm safety margins and refine procedural guidelines. Kirstein *et al*. measured intraluminal oesophageal temperatures in consecutive symptomatic patients undergoing first-time PFA, where 8 pulse trains (2 kV/2.5 s, bipolar, biphasic, ×4 basket/flower configuration each) were delivered to each pulmonary vein, and two extra pulse trains per pulmonary vein in flower configuration were added for wide antral circumferential ablation. Continuous intraluminal oesophageal temperature was monitored with a 12-pole temperature probe. The authors found that (i) median oesophageal temperature change was statistically significant and increased by 0.8 ± 0.6°C, *P* < 0.001, (ii) an oesophageal temperature increase ≥ 1°C was observed in 10/43 (23%) patients, and (iii) the highest oesophageal temperature measured was 40.3°C.^6^ Verma *et al*.^7^ measured the temperature profile of focal point, monopolar biphasic PFA on perfused thigh muscle of swine. Pulsed field ablation lesions were performed with three compatible ablation catheters, and temperature changes in the tissue were measured using fluoroptic temperature probes inserted at the muscle surface and at 3 and 7 mm below the surface. Temperatures were recorded continuously at baseline, during delivery, and after ablation. The maximum average temperature rise for PFA was 7.6°C, 2.8°C, and 0.9°C at the surface, 3 mm depth, and 7 mm depth, respectively. The temperature rise was dose dependent, with lower energy settings yielding less temperature rise.^7^ Notably, in these studies, the thermal effects resulted in luminal oesophageal temperatures above the thresholds typically used to cease radiofrequency energy delivery to avoid development of oesophageal injury.

## Conclusions

Thermal effects induced by PFA depend on total energy deposited, of which peak voltage is an important component, suggesting that current commercially available systems appear to have the potential to induce collateral thermal injury, particularly with repeated applications of pulsed field energy. Although real-world clinical outcomes suggest lower risks of oesophageal injury compared with radiofrequency ablation, the potential for thermal injury with PFA remains.

## Data Availability

Data available upon request.
